# Challenges of interpreting epidemiologic surveillance pertussis data with changing diagnostic and immunization practices: the case of the state of São Paulo, Brazil

**DOI:** 10.1186/s12879-018-3004-1

**Published:** 2018-03-13

**Authors:** Eder Gatti Fernandes, Ana Marli Christovam Sartori, Patrícia Coelho de Soárez, Telma Regina M. P. Carvalhanas, Marcela Rodrigues, Hillegonda Maria Dutilh Novaes

**Affiliations:** 1 Departamento de Medicina Preventiva, Faculdade de Medicina da Universidade de São Paulo, Av. Dr. Arnaldo, 455 2°andar, sala 2228, CEP, São Paulo, SP 01246-903 Brazil; 2 Divisão de Imunização, Centro de Vigilância Epidemiológica “Prof. Alexandre Vranjac”, Coordenadoria de Controle de Doenças da Secretaria de Estado da Saúde de São Paulo, Av. Dr Arnaldo, 351, 6° andar, Pacaembu, São Paulo, SP 01246-000 Brazil; 3 Departamento de Moléstias Infecciosas e Parasitárias, Faculdade de Medicina da Universidade de São Paulo, Av. Dr. Enéas de Carvalho Aguiar, 255, ICHC,4°andar, sala 4028 Cerqueira César, São Paulo, SP 05403-000 Brazil; 4 Divisão de Doenças de Transmissão Respiratória, Centro de Vigilância Epidemiológica “Prof. Alexandre Vranjac”, Coordenadoria de Controle de Doenças da Secretaria de Estado da Saúde de São Paulo, Av. Dr Arnaldo, 351, 6° andar, Pacaembu, São Paulo, SO 01246-000 Brazil

**Keywords:** Epidemiological surveillance, Whooping cough, Epidemics, Pertussis vaccine

## Abstract

**Background:**

A significant increase in pertussis incidence occurred in Brazil, from 2011 to 2014, despite high coverage of childhood immunization with whole-cell-pertussis (wP) containing vaccines. This study presents pertussis surveillance data from São Paulo state and discusses the challenges to interpret them considering pertussis cyclic epidemic behavior, the introduction of new diagnostic techniques and new vaccination strategies, and enhanced disease awareness during epidemics.

**Methods:**

Observational study including pertussis cases reported to the Surveillance System in São Paulo state, from January 2001 to December 2015. Pertussis cases data were retrieved from the National Notifiable Diseases Information System (SINAN) website and from São Paulo state Epidemiological Surveillance Center (CVE/SP) database. Vaccination coverage and homogeneity data were collected from the Unified Health System Department of Informatics (DATASUS). We presented cases distribution by year, age group and diagnostic criteria and calculated pertussis incidence rates. The proportions of cases among different age groups were compared using chi-square test for trend.

**Results:**

Infants less than 1 year of age were the most affected during the whole period, but the proportions of cases in this age group had a significant decreasing trend, with significant increase in the proportions of cases reported among older age groups (1–4, 5–10 and ≥20 years). Cases among infants aged less than 6 months represented ≥90% of all cases in children less than 1 year of age in all but 2 years (2012 and 2015). A non-significant decrease in the proportion of cases among infants aged < 2 months was observed in parallel to a significant increase in the proportion of cases in infants aged 6–11 months.

**Conclusions:**

A pertussis outbreak has occurred in a state with universal use of wP vaccine. The disease cyclic behavior has probably had a major role in the increased incidence rates registered in São Paulo state, from 2011 to 2014, as well as in the decreased incidence in 2015. Maternal vaccination cannot explain the drop in the number of cases among all age groups, in 2015, as herd protection is not expected, but may have had an impact on the number of cases in infants aged < 2 months.

## Background

Pertussis is a highly contagious respiratory disease mainly caused by *Bordetella pertussis* [1, 2]. It causes uncontrollable violent coughing for long periods, most commonly affects infants and young children and can be fatal, especially in infants up to 6 months of age [1, 2]. The introduction of childhood immunization with pertussis containing vaccines led to important reduction in pertussis incidence in countries that achieved high vaccine coverage, even though the disease has never disappeared [2, 3].

A global reemergence of pertussis has been observed in the last 20 years, in spite of sustained high vaccine coverage, particularly but not exclusively, in countries that adopted acellular pertussis vaccines in routine childhood immunization [3–7]. Hypotheses to explain this reemergence are post-vaccination waning immunity; reduced effectiveness of acellular vaccines; implementation of molecular methods for diagnosis; improvement of surveillance systems; enhanced disease awareness; and genetic changes in the pathogen [4, 8].

In Brazil, pertussis is a notifiable disease since 1973, and the clinical, epidemiologic and laboratory criteria used for case classification are defined by the national public health surveillance system and updated when necessary [9]. State and municipal levels may introduce additional measures to improve surveillance and assistance to pertussis cases. This variability in criteria and surveillance routines contributes to problems in temporal and spatial comparisons of pertussis incidence [4].

In the early 1980s, more than 40,000 cases were notified in Brazil each year and the annual incidence rate was > 30/100,000 inhabitants [9]. Brazilian childhood routine immunization schedule includes 3 doses of the diphtheria-tetanus-whole-cell pertussis + *Haemophilus influenzae* b + hepatitis B (DTwP-Hib-HBV) pentavalent vaccine given at 2, 4 and 6 months of age, followed by two boosters doses of DTwP at 15 and 48 months of age. In the public Unified Health System (*Sistema Único de Saúde*, SUS), the pediatric diphtheria-tetanus-acellular pertussis (DTaP) is available only for high-risk children (preterm infants, children with severe neurological or cardiac conditions and those who have previously had severe adverse events following DTwP) [10]. DTap is also available in private immunization clinics. The number of pertussis cases decreased during the 1990s, with the increase in the DTwP vaccine coverage nationally, but never disappeared, and a change in pertussis epidemiology was observed, with longer intervals between outbreaks (3–4 years) and an increase of cases in older children and adults, less detected by the surveillance systems [3, 9, 11].

A significant increase in pertussis incidence rates has been observed in Brazil, from 2011 to 2014, with the highest rates in infants younger than 3 months, and a concomitant increase in pertussis-related infant deaths, which led to a review of control strategies [3, 7]. In November 2014, the Brazilian Ministry of Health recommended an adult diphtheria-tetanus-acellular pertussis vaccine (Tdap) to all pregnant women [7]. Infant protection following maternal vaccination is a combination of the direct effect of transplacental antibody transfer from mother to infant, and the indirect effect of protecting the mother, potentially reducing household transmission and preventing infant infection [12–14].

This study presents pertussis surveillance data from São Paulo State, in the Southeast of Brazil, from 2001 to 2015, and discusses the challenges to their interpretation when considering the combined impact of cyclic epidemic variation, the introduction of new diagnostic techniques and new vaccination strategies, and enhanced disease awareness during the recent epidemic.

## Methods

### Study design

This is an observational study, including suspected and confirmed pertussis cases reported to the Surveillance System in the São Paulo state, from January 2001 to December 2015.

### Setting

São Paulo is the wealthiest of 27 Brazilian states and Federal District, being responsible for one-third of Brazil’s gross domestic product, and has a population of 43.6 million (one-fifth of Brazilian population). Its annual birth cohort is approximately 600,000, representing 1/6 of the Brazilian annual births [15].

### Source of information

In Brazil, all cases of pertussis should be reported to the National Notifiable Diseases Information System (Sistema de Informação de Agravos de Notificação, SINAN). All health professionals of both public and private health services must report a suspected case of pertussis. Nasopharyngeal swabs may be collected and sent to a reference laboratory for PCR and/or culture. After the reporting, the case and its home contacts are investigated by the municipal surveillance service. Symptomatic communicants or communicants who have contact with vulnerable persons (immunocompromised, infants, etc.) should receive chemoprophylaxis (manual de 2014, e nota técnica). The SINAN records data on location, demographic characteristics, clinical signs and symptoms, hospitalization, outcome (cure or death) and vaccination status of cases [9]. SINAN anonymous data are freely available on line.

Pertussis cases are confirmed by: I. Clinical criteria – a person with cough for 14 days or more (10 days for infants under 6 months of age) associated with two or more of the following signs and symptoms: paroxysmal cough, inspiratory gasp (whoop), and/or vomiting after coughing; II. Laboratory criteria - all individuals who met the definition of a suspected case of pertussis and have *B. pertussis* isolated in culture or identified by real time polymerase chain reaction (RT-PCR); and III. Clinical-epidemiological criteria - all individuals who met the definition of a suspected case and who had contact with a case of pertussis confirmed by laboratory testing, during the transmission period.

Data on confirmed cases that occurred before 2007 were retrieved from SINAN website. Data not freely accessible, such as suspected cases, were made available for this study by the São Paulo State Epidemiological Surveillance Center (CVE-SP). Cases were stratified by age, diagnostic confirmation criteria, and health outcome (death or cure). Information on vaccination coverage and homogeneity were collected from the Department of Informatics of the Unified Health System (DATASUS) website [16]. The Brazilian National Immunization Program (PNI) estimates the administrative vaccine coverage as the number of third dose of pediatric pertussis-containing vaccines administered divided by the number of children in the target age group multiplied by 100. Homogeneity is defined by the number of municipalities with vaccine coverage ≥95% divided by the total number of municipalities.

Population data from Brazilian Institute of Geography and Statistics [15] were used to calculate pertussis incidence before 2011, while population data from São Paulo State *Fundação Sistema Estadual de Análise de Dados*, SEADE, were used to calculate pertussis incidence in 2011 and after [17].

### Statistical analysis

Descriptive statistics are presented, with the distribution of cases by year, age group, and diagnostic criteria. The case fatality rates were presented as the proportion (%) of deaths among pertussis cases. The mean incidence rates of pertussis were calculated as the number of confirmed pertussis cases / São Paulo State population in a specific year X 100,000 inhabitants. We compared the proportion of cases among different age groups from 2001 to 2015 using chi-square test for trend with a statistical significance of 5%. Analyses were performed with EpiInfo 7.1.2.0 (Centers for Disease Control and Prevention, Atlanta, Georgia, USA).

### Ethics

The study was approved by the Research Ethics Committee of Medical Scholl of São Paulo University, license number 1.073.607.

## Results

**Fig. 1:**
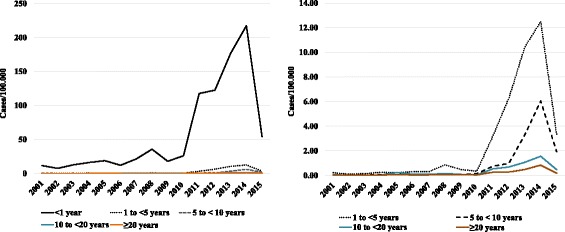
Pertussis incidence by age groups, from 2011 to 2014, São Paulo State, Brazil

In São Paulo state, pertussis incidence ranged from 0.15 to 0.76 per 100,000 inhabitants from 2001 to 2010. A considerable increase was observed in 2011, with 2.20 per 100,000, rising to 2.48 per 100,000 in 2012, 3.81 per 100,000 in 2013, and 5.06 per 100,000 in 2014. In 2015, the incidence dropped to 1.28 per 100,000. As shown in Fig. 1, pertussis incidence increased in all age groups from 2011 to 2014.

High pertussis vaccination coverage (≥95%) among infants was sustained during the entire period, except in 2012, when DTP_3_ coverage was 94%. The homogeneity was under 70% in 11 of 15 evaluated years. The lowest homogeneity observed was 45.4%, in 2012. In 2015, the first year of maternal dTpa vaccination, vaccine coverage among pregnant women was 61% in São Paulo State.

All pertussis deaths reported from 2001 to 2015 occurred among children aged less than 1 year. The number of deaths ranged from 0, in 2002, to 50, in 2013, and the higher case fatality rate was observed in 2001 (4.4%). Case fatality rates among infants aged less than 1 year were 2.79%, during the low incidence period (2001–2009) and 3.65% in the epidemic period (2011–2015).

Table 1 presents the number and proportions of pertussis cases by age group from 2001 to 2015. Infants less than 1 year of age were the most affected age group during the whole period. They represented 85.7% of all cases in 2010. This proportion dropped to 78.4% in 2011 and continued decreasing in the next years (72.5%, in 2012, 66.9%, in 2013, 63.0%, in 2014, and 61.4%, in 2015). The proportion of cases in children less than 1 year of age had a significant decreasing trend (Table 1, χ^2^ = 148.24, *p* < 0.01) with significant increases in the proportion of cases reported among older age groups (1 to 4, 5 to 10 and ≥20 years, χ^2^ = 68.20, 63.05, and 14.22, respectively, *p* < 0.01).

**Table 1:** Number and proportion of confirmed pertussis cases by age group from 2001 to 2015, São Paulo State, Brazil

Year	< 1 year	Percent	1 to < 5 years	Percent	5 to < 10 years	Percent	10 to < 20 years	Percent	≥20 years	Percent	Total	Percent
2001	73	(81.11)	6	(6.67)	3	(3.33)	4	(4.44)	4	(4.44)	90	(100)
2002	46	(83.64)	3	(5.45)	–	–	2	(3.64)	4	(7.27)	55	(100)
2003	77	(89.53)	4	(4.65)	1	(1.16)	2	(2.33)	2	(2.33)	86	(100)
2004	99	(86.09)	7	(6.09)	2	(1.74)	2	(1.74)	5	(4.35)	115	(100)
2005	116	(68.24)	6	(3.53)	8	(4.71)	17	(10.00)	23	(13.53)	170	(100)
2006	72	(75.00)	8	(8.33)	2	(2.08)	6	(6.25)	8	(8.33)	96	(100)
2007	518	(80.31)	38	(5.89)	13	(2.02)	23	(3.57)	53	(8.22)	645	(100)
2008	214	(80.15)	22	(8.24)	4	(1.50)	10	(3.75)	17	(6.37)	267	(100)
2009	107	(74.31)	12	(8.33)	2	(1.39)	6	(4.17)	17	(11.81)	144	(100)
2010	156	(85.71)	7	(3.85)	4	(2.20)	2	(1.10)	13	(7.14)	182	(100)
2011	718	(78.38)	67	(7.31)	21	(2.29)	35	(3.82)	75	(8.19)	916	(100)
2012	755	(72.53)	135	(12.97)	27	(2.59)	44	(4.23)	80	(7.68)	1041	(100)
2013	1079	(66.89)	231	(14.32)	90	(5.58)	68	(4.22)	145	(8.99)	1613	(100)
2014	1359	(62.95)	282	(13.06)	163	(7.55)	98	(4.54)	257	(11.90)	2159	(100)
2015	339	(61.41)	77	(13.95)	51	(9.24)	29	(5.25)	56	(10.14)	552	(100)
χ^2^		148.24		68.20		63.05		0.31		14.22		
*p*		< 0.01		< 0.01		< 0.01		0.57		< 0.01		

As shown in Table 2, the proportion of cases among infants aged less than 2 months and from 2 to 5 months represented 90% or more of all cases among infants aged less than 1 year in all but 2 years (2012 and 2015), with variation but no significant trend. However, an increasing trend was observed for infants aged from 6 to 11 months (χ^2^ = 12.03, *p* < 0.01).

**Table 2:** Number and proportion of pertussis cases aged < 1 year-old by age group, from 2001 to 2015, São Paulo State, Brazil

Year	< 2 months	Percent	2 to < 6 months	Percent	6 months to < 1 year-old	Percent	Total	Percent
2001	28	(38.36)	40	(54.79)	5	(6.85)	73	(100)
2002	18	(39.13)	26	(56.52)	2	(4.35)	46	(100)
2003	28	(36.36)	47	(61.04)	2	(2.60)	77	(100)
2004	37	(37.37)	57	(57.58)	5	(5.05)	99	(100)
2005	45	(38.79)	65	(56.03)	6	(5.17)	116	(100)
2006	35	(48.61)	35	(48.61)	2	(2.78)	72	(100)
2007	48	(37.80)	71	(55.91)	8	(6.30)	127	(100)
2008	88	(41.12)	111	(51.87)	15	(7.01)	214	(100)
2009	43	(40.19)	60	(56.07)	4	(3.74)	107	(100)
2010	61	(39.10)	81	(51.92)	14	(8.97)	156	(100)
2011	249	(34.68)	410	(57.10)	59	(8.22)	718	(100)
2012	295	(39.07)	376	(49.80)	84	(11.13)	755	(100)
2013	400	(37.07)	588	(54.49)	91	(8.43)	1079	(100)
2014	509	(37.45)	736	(54.16)	114	(8.39)	1359	(100)
2015	105	(30.97)	195	(57.52)	39	(11.50)	339	(100)
χ^2^		2.31		0.22		12.03		
*p*		0.13		0.64		< 0.01		

Table 3 presents the distribution of suspected and confirmed cases by confirmation criteria, from 2007 to 2015. The laboratory confirmation rate among confirmed cases increased from 2007 to 2015 (Table 3, χ^2^ = 9.50, *p* < 0.01). The laboratory confirmation rate increased from 62.5%, in 2009, to 83.97% in 2010, the first year after PCR introduction as a laboratory diagnostic tool. However, the proportion of confirmed cases (all criteria) among suspected cases decreased during the years (χ^2^ = 181.25, *p* < 0.01). The year 2015 presented the lowest proportion of confirmed cases among the suspected cases.

**Table 3:** Suspected and confirmed cases distributed by confirmation criteria, from 2007 to 2015, in São Paulo State, Brazil

	Confirmed cases					
Year	Clinical or Clinical epidemiological criteria	Laboratory criteria	Total	Suspected cases	% of confirmed among suspected cases	% of laboratory confirmed among confirmed cases
2007	68	88	156	645	24.19	56,41
2008	99	168	267	1111	24.03	62,92
2009	54	90	144	641	22.46	62,50
2010	31	151	182	692	26.30	83.97
2011	119	797	916	2787	32.87	87,01
2012	168	873	1041	4962	21.00	83,86
2013	304	1309	1613	7801	20.68	81,15
2014	434	1725	2159	10,258	21.05	79,89
2015	183	369	552	4385	12.59	66,84
χ^2^					181.25	9.5
*p*					< 0.01	< 0.01

## Discussion

A considerable increase in the pertussis incidence was observed in São Paulo State, from 2011 to 2014, in spite of high DTP_3_ vaccination coverage among children, similar to what was observed in other countries that adopt acellular pertussis vaccines. In 2015, pertussis incidence dropped to a pre-epidemic level [4–6, 18].

The global resurgence of pertussis has led to intense discussion of its causes. The predominant hypothesis to explain recent pertussis epidemiology changes are the lower effectiveness and shorter duration of protection of acellular pertussis pediatric vaccines as compared to the whole-cell vaccines [4, 8]. The shorter immunity in those vaccinated with acellular vaccines make them susceptible to the infection at a younger age [4, 8]. The waning immunity increases the number of susceptibles and, consequently, increases the circulation of *B. pertussis* among adolescents and adults, who are the source of infection for the not fully immunized infants [4, 19, 20]. However, in Brazil, the whole-cell pertussis containing vaccines are still used as part of the National Immunization Program. The proportion of children in São Paulo state who have been immunized with acellular pertussis vaccines is not known, but is considered to be very small. Other Latin American countries, such as Argentina, Colombia and Chile also reported pertussis resurgence despite adopting the whole-cell vaccine in their official vaccination schedule [3]. Thus, the increase in the pertussis incidence observed in these countries in recent years must have other explanations.

From 2006 to 2012, the proportion of Brazilian municipalities with a 95% DTP coverage decreased from 83% to 55%, with no homogeneous coverage throughout the country [3]. A study that analyzed data for the whole country found a correlation of lower vaccine coverage with higher pertussis incidence in the pre-epidemic years (2007–2011), but this correlation was not observed in the epidemic years (2012–2014) [7]. Correlation of vaccine coverage and pertussis incidence was not analyzed for the São Paulo state, which had high DTP coverage with low homogeneity in most years.

Cyclic epidemic peaks of pertussis have been observed in many countries. The interval between these peaks varies from one study to another, suggesting that they occur every two to 5 years. Brazilian data show increases in the years 1997–1998, 2004–2005 and 2007–2008 [7]. The cyclic behavior of the disease has probably had a major role in the increased incidence rates registered in São Paulo state from 2011 to 2014.

Infants aged less than 1 year were the most affected, particularly infants younger than 2 months, who had not received any vaccine dose. This age group more frequently presents the typical clinical manifestation of the disease, concentrating severe cases, with higher hospitalization and case-fatality rates, and is a reliable indicator of overall transmission activity [21–23]. On the other hand, older children, adolescents and adults usually have milder symptoms, similar to viral infections, making pertussis diagnosis difficult [21, 24]. We observed an increase in pertussis incidence rates among all age groups during the epidemic years (2011 to 2014). However, the increase was proportionally higher among older age groups when compared to infants.

This may have been related to increased awareness of pertussis among health professionals, improved diagnostic sensitivity with the introduction of PCR and better case reporting, not to changes in the age distribution of the disease. Cases among older age groups are often underdiagnosed due to the absence of typical symptoms [4, 21]. Underreporting is a concern in any surveillance system, but the low proportion of confirmed pertussis cases among the suspected cases in 2015 suggests that increased awareness was still present.

After PCR introduction in pertussis diagnosis in São Paulo State, in 2009, the proportion of confirmed cases among the suspects increased in 2010 and 2011, returned to previous levels in 2012 to 2014, and decreased significantly in 2015. The laboratorial confirmation rate among confirmed cases increased after 2010 and decreased significantly in 2015 (Table 2). The new diagnostic method may have contributed to the increased specificity of pertussis cases detection from 2011 to 2014. The availability of a new diagnostic method may have stimulated health professionals to think about the disease, increasing their awareness. But, it seems that PCR introduction has not influenced the overall epidemiological surveillance sensitivity. Pertussis incidence dropped among all age groups in 2015, preserving the proportion of cases in children aged less than 1 year and older age groups observed during the epidemic years (2011 to 2014).

Vaccination of pregnant women with Tdap was introduced in November 2014, and vaccine coverage reached 61% in São Paulo State in 2015. A case-control study estimated that maternal vaccination with Tdap had an effectiveness of 93% in protecting the infants [12]. An assessment of maternal vaccination program in England showed vaccine effectiveness of 91% (95% CI, 84 to 95%) among infants aged less than 3 months [13]. In São Paulo, a non-significant decrease in the proportion of cases among infants aged less than 2 months was observed in 2015, in comparison to a significant increase of the proportion of cases in infants aged 6 to 11 months, suggesting an impact of the maternal vaccination program on young infants (Table 3). However, the maternal vaccination program cannot explain the drop in the number of cases among all age groups, as herd protection is not expected.

Studies with mathematical models indicate pertussis resurgence in England and US was caused by lower efficacy and duration of protection of aP as compared to wP vaccine [25]. The World Health Organization recommends that countries currently using wP containing vaccines for primary immunization should continue to use them, and a switch from wP to aP should be considered only when additional strategies to control infant disease such as maternal immunization can be assured [2].

Pertussis case fatality rates ranged without any specific pattern from 2001 to 2015. The vaccination status of reported cases was not show because the proportions of unavailable information on the vaccination status ranged from 16.6% for infants aged less than 2 months to 68.5% for persons aged ≥20 years. There were inconsistencies in the vaccination status data retrieved from the notifiable diseases information system (SINAN).

## Conclusions

The data presented, based on epidemiological surveillance and straightforward descriptive analysis, helped to identify the multiple elements that may have contributed to changes in pertussis epidemiology observed in a state with universal use of wP vaccines. It suggests that the disease cyclic behavior probably had a major role in the 4 years of increased incidence followed by decrease of pertussis incidence observed in 2015. Awareness of the disease, improved diagnostic sensitivity with the introduction of PCR and better case reporting may have influenced, although not significantly the epidemiologic scenario. This type of analysis is based only on epidemiologic surveillance expertise and can be useful in the initial interpretation of the impact of new technologies on pertussis epidemiology.

## Abbreviations

CVE/SP: São Paulo state Epidemiological Surveillance Center
DATASUS: Unified Health System Department of Informatics
DTaP: Pediatric diphtheria-tetanus-acellular pertussis
DTwP-Hib-HBV: Diphtheria-tetanus-whole-cell pertussis + *Haemophilus influenzae* b + hepatitis B
IBGE: Brazilian Institute of Geography and Statistics
PNI: Brazilian National Immunization Program
RT-PCR: Real time polymerase chain reaction
SEADE: *Fundação Sistema Estadual de Análise de Dados*
SINAN: National Notifiable Diseases Information System
SUS: Unified Health System
Tdap: Adult diphtheria-tetanus-acellular pertussis vaccine
wP: Whole-cell-pertussis vaccine
